# 3-(4-Methoxy­benz­yl)-1-benzothio­phene

**DOI:** 10.1107/S1600536810018866

**Published:** 2010-05-26

**Authors:** B. Gunasekaran, V. Dhayalan, A. K. Mohanakrishnan, G. Chakkaravarthi, V. Manivannan

**Affiliations:** a Department of Physics, AMET University, Kanathur, Chennai 603 112, India; b Department of Organic Chemistry, University of Madras, Guindy Campus, Chennai 600 025, India; c Department of Physics, CPCL Polytechnic College, Chennai 600 068, India; d Department of Research and Development, PRIST University, Vallam, Thanjavur 613 403, Tamil Nadu, India

## Abstract

In the title compound, C_16_H_14_OS, the dihedral angle between the benzothio­phene ring system and the benzene ring is 72.41 (12)°. A weak inter­molecular C—H⋯π inter­action from the benzene ring to the benzothio­phene ring system is observed in the crystal structure.

## Related literature

For the biological activity of thio­phene derivatives, see: Bonini *et al.* (2005 ▶); Brault *et al.* (2005 ▶); Isloora *et al.* (2010 ▶). For related structures, see: Gunasekaran *et al.* (2009 ▶); Umadevi *et al.* (2009 ▶). For bond-length data, see: Allen *et al.* (1987 ▶).
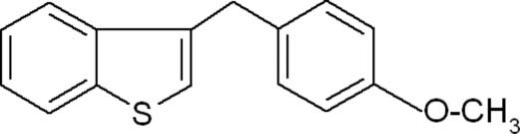


## Experimental

### 

#### Crystal data

C_16_H_14_OS
*M* *_r_* = 254.33
Monoclinic, 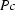

*a* = 8.0158 (6) Å
*b* = 10.8230 (9) Å
*c* = 8.1219 (6) Å
β = 112.563 (4)°
*V* = 650.68 (9) Å^3^
*Z* = 2
Mo *K*α radiation
μ = 0.23 mm^−1^
*T* = 295 K
0.25 × 0.20 × 0.20 mm

#### Data collection

Bruker SMART APEXII CCD diffractometer
Absorption correction: multi-scan (*SADABS*; Sheldrick, 1996 ▶) *T*_min_ = 0.946, *T*_max_ = 0.954
6033 measured reflections
2946 independent reflections
2721 reflections with *I* > 2σ(*I*)
*R*_int_ = 0.171

#### Refinement

*R*[*F* ^2^ > 2σ(*F* ^2^)] = 0.066
*wR*(*F* ^2^) = 0.176
*S* = 1.06
2946 reflections
164 parameters
2 restraints
H-atom parameters constrained
Δρ_max_ = 0.35 e Å^−3^
Δρ_min_ = −0.48 e Å^−3^
Absolute structure: Flack (1983 ▶), 1337 Friedel pairs
Flack parameter: −0.04 (11)

### 

Data collection: *APEX2* (Bruker, 2004 ▶); cell refinement: *SAINT* (Bruker, 2004 ▶); data reduction: *SAINT*; program(s) used to solve structure: *SHELXS97* (Sheldrick, 2008 ▶); program(s) used to refine structure: *SHELXL97* (Sheldrick, 2008 ▶); molecular graphics: *PLATON* (Spek, 2009 ▶); software used to prepare material for publication: *SHELXL97*.

## Supplementary Material

Crystal structure: contains datablocks global, I. DOI: 10.1107/S1600536810018866/is2551sup1.cif

Structure factors: contains datablocks I. DOI: 10.1107/S1600536810018866/is2551Isup2.hkl

Additional supplementary materials:  crystallographic information; 3D view; checkCIF report

## Figures and Tables

**Table 1:** Hydrogen-bond geometry (Å, °) *Cg*1 is the centroid of the C1–C6 ring.

*D*—H⋯*A*	*D*—H	H⋯*A*	*D*⋯*A*	*D*—H⋯*A*
C14—H14⋯*Cg*^i^	0.93	2.83	3.617 (2)	143

Symmetry code: (i) 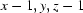.

**Fig. 1.**
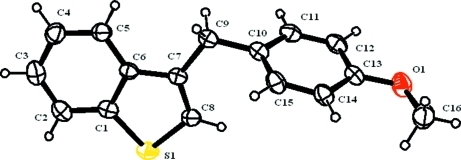
The molecular structure of (I), with atom labels and 30% probability displacement ellipsoids for non-H atoms.

## supplementary crystallographic information

### Comment

Thiophene derivatives exhibit anti-HIV PR inhibitors (Bonini *et al.*, 2005) and anti-breast cancer (Brault *et al.*, 2005) activities. In addition, some of the benzo[b]thiophene derivatives shows significant antimicrobial and anti-inflammatory activities (Isloora *et al.*, 2010).

The geometric parameters of the title molecule (Fig. 1) agree well with reported similar structures (Gunasekaran *et al.*, 2009; Umadevi *et al.*, 2009). The dihedral angle between the two benzene rings is 71.93 (8)°. The C1—S1 and C8—S1 bond distances are 1.738 (3) and 1.734 (3) Å respectively, which are comparable to the literature value of 1.712 (2) Å (Allen *et al.*, 1987).

The crystal packing is stabilized by a weak C—H···π interaction [C14—H14···*Cg* (-1+*x*, *y*, -1+*z*), Table 1; *Cg* is the centroid of the ring defined by the atoms C1—C6] .

### Experimental

To a solution of 1-(bromomethyl)-4-methoxybenzene (0.7 g, 3.48 mmol) in dry 1,2-dichloroethane (20 ml) ZnBr_2_ (0.23 g, 1.02 mmol) and benzo[b]thiophene (0.7 g, 5.22 mmol) were added. It was then stirred at room temperature for 6 h under N_2_ atmosphere. The solvent was removed and the residue was quenched with ice-water (50 ml) containing 1 ml of conc. HCl, extracted with chloroform (2 × 10 ml) and dried (Na_2_SO_4_). Removal of solvent followed by column chromatographic purification (n-hexane/ethyl acetate 94:6) afforded the product as a colourless crystal.

### Refinement

H atoms were positioned geometrically and refined using riding model with C—H = 0.93 Å and *U*_iso_(H) = 1.2*U*_eq_(C) for aromatic C—H, C—H = 0.97 Å and *U*_iso_(H) = 1.2*U*_eq_(C) for CH_2_, C—H = 0.96 Å and *U*_iso_(H) = 1.5*U*_eq_(C) for CH_3_.

### Crystal data

**Table:**

C_16_H_14_OS	*F*(000) = 268
*M**_r_* = 254.33	*D*_x_ = 1.298 Mg m^−^^3^
Monoclinic, *P**c*	Mo *K*α radiation, λ = 0.71073 Å
Hall symbol: P -2yc	Cell parameters from 4241 reflections
*a* = 8.0158 (6) Å	θ = 2.7–28.3°
*b* = 10.8230 (9) Å	µ = 0.23 mm^−^^1^
*c* = 8.1219 (6) Å	*T* = 295 K
β = 112.563 (4)°	Block, colourless
*V* = 650.68 (9) Å^3^	0.25 × 0.20 × 0.20 mm
*Z* = 2	

### Data collection

**Table:**

Bruker SMART APEXII CCD diffractometer	2946 independent reflections
Radiation source: fine-focus sealed tube	2721 reflections with *I* > 2σ(*I*)
graphite	*R*_int_ = 0.171
Detector resolution: 0 pixels mm^-1^	θ_max_ = 28.3°, θ_min_ = 1.9°
ω and φ scans	*h* = −10→9
Absorption correction: multi-scan (*SADABS*; Sheldrick, 1996)	*k* = −12→14
*T*_min_ = 0.946, *T*_max_ = 0.954	*l* = −10→10
6033 measured reflections	

### Refinement

**Table:**

Refinement on *F*^2^	Secondary atom site location: difference Fourier map
Least-squares matrix: full	Hydrogen site location: inferred from neighbouring sites
*R*[*F*^2^ > 2σ(*F*^2^)] = 0.066	H-atom parameters constrained
*wR*(*F*^2^) = 0.176	*w* = 1/[σ^2^(*F*_o_^2^) + (0.1211*P*)^2^ + 0.025*P*] where *P* = (*F*_o_^2^ + 2*F*_c_^2^)/3
*S* = 1.06	(Δ/σ)_max_ < 0.001
2946 reflections	Δρ_max_ = 0.35 e Å^−^^3^
164 parameters	Δρ_min_ = −0.48 e Å^−^^3^
2 restraints	Absolute structure: Flack (1983), 1337 Friedel pairs
Primary atom site location: structure-invariant direct methods	Flack parameter: −0.04 (11)

### Fractional atomic coordinates and isotropic or equivalent isotropic displacement parameters (Å^2^)

**Table:**

	*x*	*y*	*z*	*U*_iso_*/*U*_eq_	
C1	0.9043 (4)	0.0721 (3)	0.6766 (3)	0.0493 (6)	
C2	1.0778 (5)	0.0242 (3)	0.7621 (4)	0.0607 (7)	
H2	1.0969	−0.0510	0.8219	0.073*	
C3	1.2203 (5)	0.0925 (4)	0.7547 (5)	0.0671 (9)	
H3	1.3374	0.0627	0.8111	0.081*	
C4	1.1926 (5)	0.2048 (4)	0.6648 (5)	0.0616 (7)	
H4	1.2912	0.2494	0.6634	0.074*	
C5	1.0204 (4)	0.2504 (3)	0.5777 (4)	0.0513 (6)	
H5	1.0025	0.3250	0.5165	0.062*	
C6	0.8728 (3)	0.1837 (2)	0.5821 (3)	0.0423 (5)	
C7	0.6844 (3)	0.2155 (2)	0.5029 (3)	0.0433 (5)	
C8	0.5818 (4)	0.1302 (3)	0.5405 (4)	0.0488 (5)	
H8	0.4568	0.1362	0.5004	0.059*	
C9	0.6202 (4)	0.3311 (3)	0.3944 (4)	0.0557 (6)	
H9A	0.6662	0.3315	0.3000	0.067*	
H9B	0.6716	0.4018	0.4703	0.067*	
C10	0.4183 (4)	0.3466 (3)	0.3119 (3)	0.0497 (6)	
C11	0.3305 (4)	0.4346 (3)	0.3745 (4)	0.0532 (6)	
H11	0.3975	0.4844	0.4704	0.064*	
C12	0.1454 (4)	0.4492 (3)	0.2967 (4)	0.0542 (6)	
H12	0.0899	0.5101	0.3387	0.065*	
C13	0.0417 (4)	0.3743 (3)	0.1570 (3)	0.0470 (6)	
C14	0.1274 (4)	0.2862 (3)	0.0923 (3)	0.0517 (6)	
H14	0.0605	0.2357	−0.0028	0.062*	
C15	0.3130 (4)	0.2744 (3)	0.1708 (4)	0.0563 (7)	
H15	0.3690	0.2153	0.1265	0.068*	
C16	−0.2496 (5)	0.3249 (5)	−0.0572 (6)	0.0793 (11)	
H16A	−0.2253	0.3485	−0.1597	0.119*	
H16B	−0.3746	0.3397	−0.0799	0.119*	
H16C	−0.2233	0.2386	−0.0332	0.119*	
O1	−0.1408 (3)	0.3948 (3)	0.0910 (3)	0.0653 (6)	
S1	0.70297 (12)	0.00836 (7)	0.67020 (11)	0.0601 (2)	

### Atomic displacement parameters (Å^2^)

**Table:**

	*U*^11^	*U*^22^	*U*^33^	*U*^12^	*U*^13^	*U*^23^
C1	0.0614 (14)	0.0440 (15)	0.0452 (11)	−0.0010 (11)	0.0235 (10)	−0.0009 (10)
C2	0.0703 (18)	0.0529 (17)	0.0552 (15)	0.0134 (14)	0.0201 (12)	0.0062 (12)
C3	0.0586 (16)	0.074 (2)	0.0644 (16)	0.0158 (15)	0.0191 (13)	−0.0042 (15)
C4	0.0546 (14)	0.0643 (18)	0.0702 (15)	−0.0037 (14)	0.0286 (12)	−0.0104 (16)
C5	0.0569 (14)	0.0447 (14)	0.0579 (13)	−0.0035 (11)	0.0283 (11)	−0.0016 (10)
C6	0.0517 (12)	0.0365 (12)	0.0417 (9)	−0.0009 (9)	0.0213 (9)	−0.0032 (8)
C7	0.0511 (11)	0.0377 (12)	0.0430 (10)	−0.0007 (10)	0.0203 (8)	−0.0007 (9)
C8	0.0549 (13)	0.0414 (13)	0.0537 (11)	−0.0035 (11)	0.0250 (10)	−0.0008 (10)
C9	0.0563 (14)	0.0430 (15)	0.0645 (14)	0.0016 (12)	0.0196 (11)	0.0102 (12)
C10	0.0580 (14)	0.0403 (13)	0.0511 (12)	0.0057 (11)	0.0211 (10)	0.0068 (10)
C11	0.0699 (16)	0.0396 (14)	0.0490 (11)	−0.0006 (12)	0.0218 (11)	−0.0037 (10)
C12	0.0717 (17)	0.0425 (14)	0.0544 (12)	0.0106 (12)	0.0309 (12)	−0.0033 (11)
C13	0.0583 (14)	0.0412 (13)	0.0447 (10)	0.0112 (10)	0.0233 (10)	0.0049 (9)
C14	0.0597 (14)	0.0473 (15)	0.0442 (10)	0.0103 (12)	0.0155 (10)	−0.0068 (10)
C15	0.0633 (16)	0.0526 (17)	0.0528 (12)	0.0161 (12)	0.0221 (11)	−0.0033 (11)
C16	0.0594 (19)	0.078 (3)	0.090 (2)	0.0062 (16)	0.0179 (17)	−0.014 (2)
O1	0.0607 (12)	0.0675 (16)	0.0672 (12)	0.0173 (11)	0.0238 (9)	−0.0048 (11)
S1	0.0723 (4)	0.0462 (4)	0.0659 (4)	−0.0059 (3)	0.0312 (3)	0.0108 (3)

### Geometric parameters (Å, °)

**Table:**

C1—C2	1.394 (5)	C9—H9A	0.9700
C1—C6	1.401 (4)	C9—H9B	0.9700
C1—S1	1.738 (3)	C10—C15	1.376 (4)
C2—C3	1.381 (6)	C10—C11	1.392 (4)
C2—H2	0.9300	C11—C12	1.380 (4)
C3—C4	1.391 (6)	C11—H11	0.9300
C3—H3	0.9300	C12—C13	1.383 (4)
C4—C5	1.379 (5)	C12—H12	0.9300
C4—H4	0.9300	C13—O1	1.369 (4)
C5—C6	1.398 (4)	C13—C14	1.391 (4)
C5—H5	0.9300	C14—C15	1.381 (4)
C6—C7	1.438 (4)	C14—H14	0.9300
C7—C8	1.347 (4)	C15—H15	0.9300
C7—C9	1.503 (4)	C16—O1	1.406 (5)
C8—S1	1.734 (3)	C16—H16A	0.9600
C8—H8	0.9300	C16—H16B	0.9600
C9—C10	1.504 (4)	C16—H16C	0.9600
			
C2—C1—C6	121.9 (3)	C10—C9—H9B	108.5
C2—C1—S1	127.2 (3)	H9A—C9—H9B	107.5
C6—C1—S1	110.9 (2)	C15—C10—C11	117.3 (3)
C3—C2—C1	117.6 (3)	C15—C10—C9	121.3 (3)
C3—C2—H2	121.2	C11—C10—C9	121.5 (3)
C1—C2—H2	121.2	C12—C11—C10	121.1 (3)
C2—C3—C4	121.5 (3)	C12—C11—H11	119.4
C2—C3—H3	119.2	C10—C11—H11	119.4
C4—C3—H3	119.2	C11—C12—C13	120.7 (2)
C5—C4—C3	120.6 (3)	C11—C12—H12	119.6
C5—C4—H4	119.7	C13—C12—H12	119.6
C3—C4—H4	119.7	O1—C13—C12	116.2 (2)
C4—C5—C6	119.5 (3)	O1—C13—C14	124.9 (3)
C4—C5—H5	120.2	C12—C13—C14	118.9 (3)
C6—C5—H5	120.2	C15—C14—C13	119.3 (3)
C5—C6—C1	118.9 (3)	C15—C14—H14	120.3
C5—C6—C7	128.2 (2)	C13—C14—H14	120.3
C1—C6—C7	112.9 (2)	C10—C15—C14	122.7 (3)
C8—C7—C6	111.2 (2)	C10—C15—H15	118.7
C8—C7—C9	127.0 (3)	C14—C15—H15	118.7
C6—C7—C9	121.8 (2)	O1—C16—H16A	109.5
C7—C8—S1	114.3 (2)	O1—C16—H16B	109.5
C7—C8—H8	122.9	H16A—C16—H16B	109.5
S1—C8—H8	122.9	O1—C16—H16C	109.5
C7—C9—C10	114.9 (2)	H16A—C16—H16C	109.5
C7—C9—H9A	108.5	H16B—C16—H16C	109.5
C10—C9—H9A	108.5	C13—O1—C16	117.8 (3)
C7—C9—H9B	108.5	C8—S1—C1	90.76 (14)
			
C6—C1—C2—C3	1.6 (4)	C6—C7—C9—C10	176.5 (2)
S1—C1—C2—C3	−178.0 (2)	C7—C9—C10—C15	−71.9 (4)
C1—C2—C3—C4	−0.3 (5)	C7—C9—C10—C11	108.3 (3)
C2—C3—C4—C5	−0.9 (5)	C15—C10—C11—C12	−0.6 (4)
C3—C4—C5—C6	0.8 (5)	C9—C10—C11—C12	179.2 (3)
C4—C5—C6—C1	0.5 (4)	C10—C11—C12—C13	1.7 (4)
C4—C5—C6—C7	179.1 (3)	C11—C12—C13—O1	179.0 (3)
C2—C1—C6—C5	−1.7 (4)	C11—C12—C13—C14	−1.9 (4)
S1—C1—C6—C5	177.95 (19)	O1—C13—C14—C15	−179.9 (3)
C2—C1—C6—C7	179.4 (3)	C12—C13—C14—C15	1.1 (4)
S1—C1—C6—C7	−0.9 (3)	C11—C10—C15—C14	−0.3 (5)
C5—C6—C7—C8	−178.0 (3)	C9—C10—C15—C14	179.9 (3)
C1—C6—C7—C8	0.8 (3)	C13—C14—C15—C10	0.0 (5)
C5—C6—C7—C9	1.7 (4)	C12—C13—O1—C16	176.7 (3)
C1—C6—C7—C9	−179.6 (2)	C14—C13—O1—C16	−2.4 (5)
C6—C7—C8—S1	−0.3 (3)	C7—C8—S1—C1	−0.2 (2)
C9—C7—C8—S1	−179.9 (2)	C2—C1—S1—C8	−179.7 (3)
C8—C7—C9—C10	−3.9 (4)	C6—C1—S1—C8	0.6 (2)

### Hydrogen-bond geometry (Å, °)

**Table:**

Cg1 is the centroid of the C1–C6 ring.

**Table:**

*D*—H···*A*	*D*—H	H···*A*	*D*···*A*	*D*—H···*A*
C14—H14···Cg^i^	0.93	2.83	3.617 (2)	143

Symmetry codes: (i) *x*−1, *y*, *z*−1.
